# A prospective cohort study protocol on sequential compression alone for the prevention of venous thromboembolism (VTE) in laparoscopic cholecystectomy

**DOI:** 10.1097/SP9.0000000000000068

**Published:** 2026-07-27

**Authors:** Chien-Tse Kao, Darren Lowen, Simone Said, Mark Tacey, Danielle Taylor, Justin M. Yeung, Russell Hodgson

**Affiliations:** a Division of Surgery, Northern Health, Epping, Australia; b Department of Anaesthesia & Perioperative Medicine, Northern Health, Epping, Australia; c Department of Critical Care, Melbourne Medical School, The University of Melbourne, Parkville, Australia; d Department of Intensive Care, Northern Health, Epping, Australia; e Department of Research, Northern Health, Epping, Australia; f Department of Medicine, Dentistry and Health Sciences, University of Melbourne, Parkville, Australia; g Department of Surgery, Western Health, Melbourne, Australia; h Department of Surgery, Western Precinct, University of Melbourne, St Albans, Australia; i Department of Surgery, University of Melbourne, Epping, Australia

**Keywords:** deep venous thrombosis, mechanical prophylaxis

## Abstract

**Background:**

Venous thromboembolism (VTE) is one of the leading causes of in-hospital mortality and is responsible for significant healthcare-related costs. However, a clear thromboprophylaxis protocol does not exist in surgery. The conventional method of thromboprophylaxis in surgery includes a combination of chemical and mechanical measures. Studies have shown that the administration of a chemical prophylaxis agent intra-operatively is associated with an increased incidence of surgical bleeding in surgeries such as laparoscopic cholecystectomy (LC). There are two common forms of mechanical prophylaxis: graduated compression stockings (GCSs) and sequential calf compression devices (SCCDs). Recent evidence has suggested several unwanted effects of GCSs, including skin breakage, the development of ulcers, and necrosis. There is a paucity of evidence investigating the use of SCCDs alone intra-operatively in VTE and surgical bleeding prevention.

**Materials and methods:**

This study reports on a multi-center, single-arm, prospective cohort study conducted across 11 hospitals, focusing on LC to standardize the operation. The aim is to recruit 5190 patients, as determined by power calculations, to investigate the incidence of clinical VTE 30 days after surgery. The hospitals involved already have policies in place to use SCCDs alone. All patients undergoing LC will be recruited prospectively, with a 30-day survey conducted to ascertain symptoms of VTE. Any reported symptoms will prompt appropriate radiological confirmation. The secondary outcome of bleeding or other complications arising from VTE prophylaxis will also be collected.

**Results:**

Ethical approval was obtained for this study. Results will be submitted for publication in a peer-reviewed journal and disseminated through participating hospitals.

**Conclusion:**

This study aims to provide information on the incidence of VTE in the LC population following the use of SCCDs only at the time of surgery and to compare it to historical controls of combined SCCDs and GCSs. The benefits include economic, safety, and environmental improvements.

## Introduction

It has been estimated that the Australian annual incidence of VTE is 0.83 per 1000 individuals and is associated with significant morbidity and health-related economic costs. In 2008, the estimated cost to the Australian economy was $1.7 billion[1]. Surgeries with a high risk of VTE include hip or knee replacement, major general, thoracic, vascular, and abdominal laparoscopic surgery^[^2–6^]^. The risk of surgically provoked VTE may be mitigated by the combined use of both chemical and mechanical thromboprophylaxis, as recommended by multiple clinical practice guidelines^[^7,8^]^. When chemical prophylaxis is given at the time of surgery, there is a documented increased risk of bleeding to the point where it is not recommended with certain types of surgery or spinal anesthesia and perhaps should only be given post-operatively in other types of surgery^[^9,10^]^. Despite this, there is no clear recommendation in guidelines regarding the timing of chemical thromboprophylaxis in relation to the operation or the type and duration of mechanical thromboprophylaxis that should be used when chemical prophylaxis is not given.

The exact role of mechanical VTE prophylaxis is not well-researched. The two common modalities used are graduated compression stockings (GCSs) and sequential calf compression devices (SCCDs). GCSs have commonly been used on patients in the ward setting, whereas SCCDs require a dedicated pump for use. In the GAPS study, the use of low molecular weight heparin (LMWH) alone was shown to be non-inferior in terms of the incidence of VTE when compared to the combined use of LMWH with GCSs[11]. This suggests that GCSs added no further protection if chemical prophylaxis is used. However, with the limitations of chemical prophylaxis during surgery, the immediate operative period still relies on mechanical prophylaxis. There is weak evidence suggesting that SCCDs are superior to the use of GCSs as a means of providing mechanical thromboprophylaxis, as evident in the 2012 ACCP guidelines and confirmed in the 2019 ASH clinical practice guidelines^[^7,8^]^. However, although there is no evidence supporting combined use, this is a common practice in operating theaters[12].

There is evidence that the use of GCSs is associated with harm (defined as skin breaks, blisters, ulcers, and necrosis)[8]. This was confirmed in the CLOTS study, with a 5% vs. 1% increase in ulceration and tissue necrosis with GCSs[13]. Additionally, the cost of GCSs is quite significant. The annual cost of the purchase and application of GCSs for surgical patients has been estimated at £63.1 million in England alone[11].

The hypothesis of this study is that SCCDs alone, as mechanical prophylaxis, are non-inferior for VTE prophylaxis when compared to combined mechanical prophylaxis in medium-risk surgery, such as LC. If true, this would eliminate the requirement for GCSs, along with the cost and complication risk, and suggest further studies for high-risk patients.

## Methods and analysis

### Aims and objectives

This study aims to determine if a standardized approach to the intra-operative use of SCCDs only will result in a non-inferior incidence of 30-day symptomatic VTE in patients undergoing either elective or emergency laparoscopic cholecystectomies compared to a reference previously published combined mechanical prophylaxis comparator group from the same mix of centers[12].

There are two main objectives of this prospective point prevalence study:

1. To determine whether the structured approach of VTE prophylaxis using SCCDs alone is non-inferior with respect to the incidence of symptomatic VTE by day 30 post-operatively when compared to the historical control.
2. To determine the incidence of surgical bleeding and SCCD-related complications.

### Study design

This is a multi-center, prospective point prevalence cohort study of patients undergoing either an emergency or elective laparoscopic cholecystectomy. The incidence of symptomatic VTE at day 30 post-surgical procedure will be compared to a previously obtained incidence of VTE at 0.3%, which was obtained for elective laparoscopic cholecystectomies only, when there was no standardization of timing of chemical thrombo-prophylaxis or the type of mechanical thromboprophylaxis used, although over two-thirds used combined mechanical thromboprophylaxis[12].

The benefits of performing this study as a cohort study are the decreased selection and participation bias, as well as an increased recruitment rate. Utilizing a hospital’s current practice also alleviates the need and cost of widescale pre-operative recruitment and consenting of patients. Given the small number of patients who develop VTE following medium-risk surgery such as LC, the cost and number of patients required for a randomized controlled trial would be impractical, and furthermore, a cohort study provides a more accurate “real-world” result. Given the simplicity of the patient survey in the study and the use of standardized datasets, no patient or public involvement was sought in the development of the protocol.

### Setting

This is designed as a multi-center study of surgical patients undergoing a laparoscopic cholecystectomy (either as an elective or an emergency procedure) across eleven hospital campuses in Australia. These hospitals have been selected as they have already adopted the study protocol (SCCDs only) as standard of care and include hospitals similar in demographic to the reference PROTECTinG study[12].

This study will utilize a surgical registrar-run research collaborative to maintain contact with the multiple sites and continue recruitment of registrars who will act as data collectors for the study. This vastly decreases the costs of the study, and if this study confirms that the addition of GCSs is superfluous, this would represent a significant cost benefit as well as environmental savings.

### Study population

The study population includes all patients undergoing either an emergency or an elective laparoscopic cholecystectomy. The total number of surgical participants required is 5190, as determined by power calculations. The sample size calculation considered a one-sided proportion test with an assumed rate of VTE of 0.24% in the study population compared to a comparative rate of 0.45%, with an alpha of 0.05 and 0.8 power. The selection of the control rate was guided by the PROTECTinG study[12], with the rate of 0.45% being considered the upper limit of a non-inferiority margin in the rate of VTE. This sample size provides a 95% confidence interval of 0.12% to 0.40% for a point estimate of 0.24% in the observed rate of VTE. This VTE rate of 0.24% represents a 20% reduction in the observed VTE incidence rate reported in the reference study. There is no minimum requirement from each of the various hospital campuses, nor is there any stratification of any of the various hospital campuses.

### Inclusion criteria

All patients who undergo a laparoscopic cholecystectomy, either as an emergency or elective procedure, at each of the hospital campuses are eligible for inclusion in this study. There is no gold standard approach for chemical and mechanical thromboprophylaxis, and the proposed intervention is one of the accepted surgical techniques for the prevention of VTE. All of the involved hospitals had already adopted SCCDs alone prior to the commencement of this study.

### Exclusion criteria

Participants will be excluded based on the following criteria:

- If they are fitted with GCSs exclusively, combined GCSs and SCCDs, or if no mechanical prophylaxis is used intra-operatively.
- A planned or unplanned conversion to open cholecystectomy.
- Under the age of 18 years.

### Primary outcomes

The primary outcome is the incidence of symptomatic VTE by day 30 post-operatively, which is proven by any imaging technique.

### Secondary outcomes

The secondary outcome is the incidence of surgical bleeding, particularly bleeding that is defined as a major bleeding event, characterized by the need for radiological or surgical control, a blood transfusion, or a drop in the baseline hemoglobin of >20 g/L.

### Study method

All patients requiring either an elective or an emergency laparoscopic cholecystectomy will be recruited for this study.

For those participants who are booked for an elective laparoscopic cholecystectomy, they will be admitted to the hospital on the day of their surgery. A sequential calf compressor device will be fitted as per the manufacturer’s specifications immediately prior to the commencement of surgery, and will be used for the duration of surgery. At the completion of surgery and immediately before the patient is transferred to the post-anesthetic care unit, the SCCDs will be removed. Chemical thromboprophylaxis will be initiated by the subcutaneous injection of 40 mg of enoxaparin, 5000 IU of dalteparin, or 5000 IU of heparin at any time at the discretion of the treating team. Subsequent doses of enoxaparin and dalteparin (if required) are given 24 hours later following the initial dose. No participant undergoing an elective operation is to have GCSs fitted either pre- or post-operatively. Participants may be discharged home on the day of their surgery at the discretion of their treating surgeon. During the operation, positioning of the patient and the intra-abdominal insufflation pressure will be at the discretion of the treating surgeon.

For those participants who are undergoing an emergency laparoscopic cholecystectomy, access to the theater may differ across the various hospital campuses. This will be controlled for by the acquisition of data pertaining to the length of time of admission to the hospital relative to the timing of their actual surgery. For these patients, it is anticipated that they will have received chemical thromboprophylaxis prior to their operation. This data will be collected and analyzed separately as a sub-analysis. There will be no restrictions on the type or timing of chemical thromboprophylaxis used.

Demographic and operative data will be collected at the end of the operation by the surgical fellow/registrar/resident who has been recruited to the study and trained under the auspices of the registrar collaborative. This process will also be supervised by the PI from each institution.

As part of this study, all patients will be contacted between days 30 and 35 post-operatively via telephone to inquire about symptoms that may be suggestive of the development of VTE, irrespective of the clinical follow-up. At this time, verbal consent will be obtained to proceed with a pro forma telephone questionnaire, and this process will occupy approximately 2–5 minutes of their time. For those participants who have symptoms suggestive of a VTE based on the Wells criteria[14], they will be invited back to their treating hospital for image confirmation of VTE, and if this is confirmed, then an anticoagulation regimen will be commenced at the discretion of the clinical team, the duration of which is guided by the latest guidelines from the Thrombosis and Haemostasis Society of Australia and New Zealand[1].

### Data collection

All data will be transcribed into a central REDCap database, accessible electronically and maintained at the lead hospital^[^15,16^]^. The defined time point for data collection will be between days 30 and 35 post-operatively, through a telephone consultation using a proforma with specific questions (Box 1) to determine if a participant has symptomatic VTE present. If the participant’s symptoms are suggestive of a VTE, they will be invited back to the treating hospital to confirm this via imaging techniques. The database will be regularly checked for completeness, and data will be randomly audited for fidelity issues.

### Safety considerations

Data to be collected are data that are already generated and captured by various hospitals as part of National Safety Standards and on-going GCP guidelines. The only data not routinely collected are the 30-day post-operative follow-up telephone calls for the determination of symptoms consistent with the development of VTE, which will then require further investigations and the possibility of an anticoagulation regimen. As this increases participants' safety and the overall quality of the surgical experience, it is felt that there is no negative impact on patient safety due to this study.

### Statistical analysis

Descriptive statistics will be prepared to provide a summary of the cohort. The incidence rate of VTE and surgical bleeding will be calculated. If the sample size allows, associations between patient and surgical factors with the primary and secondary outcomes will be analyzed using appropriate continuous or categorical statistical tests. Given the expected low incidence rate of VTE, multivariable analysis is likely to be limited to the inclusion of two variables to assess potential confounding effects. The potentially higher incidence rate of surgical bleeding will allow up to 10 variables to be included, according to sample size calculations indicated by Peduzzi *et al*[17].

Subgroup analysis will be performed on elective and emergency admission patients, as well as low, medium, and high-risk patients according to their Caprini Score[18].

### Limitations/risk of bias

The cohort design of the study limits selection and participation bias. It is not expected that the measurements of primary or secondary outcomes will pose a high risk of bias, as these outcomes are measured objectively by biochemical and radiological parameters. However, abnormal blood results, such as decreased hemoglobin count, can result from a poor blood sample or dilutional effects.

Limitations of the study include the use of a historical control, although this is partially addressed by including the same and similar hospitals between studies. Utilizing a collaborative authorship model has great advantages but also presents limitations, such as the potential for inconsistent data entry. The main limitation in terms of VTE risk is the variation in hospital policy regarding the use of chemical VTE thromboprophylaxis; however, restricting the study to hospitals with identical chemical prophylaxis policies would exclude most sites, and requiring individual participant consent and recruitment was deemed too resource-intensive and likely to introduce bias. Additionally, there was no patient or public involvement in the design of the study.

The use of a single type of operation, laparoscopic cholecystectomy, limits the heterogeneity of the operative data and, therefore, potential bias. However, the incidence of VTE in laparoscopic cholecystectomy is lower than that for other higher-risk operations (e.g., abdominal cancer resections), necessitating a large population. While abdominal laparoscopy is considered high risk for VTE, it is clearly not the highest-risk surgery. Results of this study may not be transferable to higher-risk operations but may inform and alleviate safety concerns for a future study on this population.

## Ethics and dissemination

This study will be conducted in full conformance with the principles of the “Declaration of Helsinki,” Good Clinical Practice, and within the laws and regulations of the states of Victoria, New South Wales, and South Australia. The study was approved by a local ethics committee (LRR 094/21) and registered with the Australian Clinical Trials Network (*ACTN number blinded*), where a full copy of the protocol is also stored.

Data will initially be obtained in a re-identifiable manner (hospital and UR number are recorded). This is required to enter the 30-day survey and audit of the 30-day morbidity into the patient dataset. This will also ensure data completeness by requesting missing data from the person involved in inputting that particular participant’s data into the database, and by allowing six-monthly random data checks to ensure accuracy. These data will be marked as identifiers in REDCap, and only aggregate, de-identified data will be extracted for analysis. Electronic data will be destroyed by electronic shredding seven years after publication.

The final publication will be written according to the STROCSS guidelines[19]. Dissemination will occur through participating hospitals, as well as publicly through local, national, and international conferences, and through peer-reviewed journals.

## Conclusion

Venous thromboembolism that has been provoked by major surgery is a modifiable risk factor that may depend on the timing and type of thromboprophylaxis. Despite the widespread use of multiple types of mechanical thromboprophylaxis during surgery, a time when chemoprophylaxis may not be appropriate, there are no strong recommendations regarding the type and timing of mechanical thromboprophylaxis. If the results from this study are proven to be non-inferior to the conventional method of thromboprophylaxis, this can form the basis of guidance for surgical teams on how to minimize surgically provoked VTE, bleeding, and GCS-related complications in medium VTE risk patients. The findings of this study may then also be applied to the design of studies of other higher-risk surgical procedures to determine the generalizability of mitigation of VTE risk across various surgical sub-specialities.

## Ethical approval

The study was approved by a local ethics committee (LRR 094/21) and registered with the Australian Clinical Trials Network.

## Consent

Not applicable.

## Sources of funding

Funding is as acknowledged, with a $5000 “Small Grant” through the Northern Health Foundation.

## Data Availability

Not applicable.

## Trial registration number

ACTRN12621001201864.
